# Plant Nuclear Factor Y (NF-Y) Transcription Factors: Evolving Insights into Biological Functions and Gene Expansion

**DOI:** 10.3390/ijms26010038

**Published:** 2024-12-24

**Authors:** Chamindika L. Siriwardana

**Affiliations:** Department of Science and Mathematics, Texas A&M University-Central Texas, Killeen, TX 76549, USA; c.siriwardana@tamuct.edu

**Keywords:** nuclear factor Y (NF-Y), transcription factors, plant genes

## Abstract

Gene expansion is a common phenomenon in plant transcription factor families; however, the underlying molecular mechanisms remain elusive. Examples of gene expansion in transcription factors are found in all eukaryotes. One example is plant nuclear factor Y (NF-Y) transcription factors. NF-Y is ubiquitous to eukaryotes and comprises three independent protein families: NF-YA, NF-YB, and NF-YC. While animals and fungi mostly have one of each NF-Y subunit, NF-Y is greatly expanded in plants. For example, humans have one each of NF-YA, NF-YB, and NF-YC, while the model plant Arabidopsis has ten each of NF-YA, NF-YB, and NF-YC. Our understanding of the plant NF-Y, including its biological roles, molecular mechanisms, and gene expansion, has improved over the past few years. Here we will review its biological roles and focus on studies demonstrating that NF-Y can serve as a model for plant gene expansion. These studies show that NF-Y can be classified into ancestrally related subclasses. Further, the primary structure of each NF-Y contains a conserved core domain flanked by non-conserved N- and C-termini. The non-conserved N- and C-termini, under pressure for diversifying selection, may provide clues to this gene family’s retention and functional diversification following gene duplication. In summary, this review demonstrates that NF-Y expansion has the potential to be used as a model to study the gene expansion and retention of transcription factor families.

## 1. Introduction

Gene duplication has been a subject of interest for biologists for over a century. In one of the earliest studies in 1936, Dr. Calvin Bridges published his duplication of the Bar gene in *Drosophila melanogaster* [1]. A turning point in the study of gene duplication was Dr. Susumu Ohno’s 1970 landmark book “*Evolution by Gene Duplication*”, which catalyzed the quest to understand the fate of duplicated genes [2]. Dr. Ohno hypothesized that the extra copies resulting from gene duplications allow the retention of mutations, ultimately providing raw material for the evolution of new phenotypes. However, in nature, deleterious mutations with negative impacts and a subsequent loss of function are more common than beneficial mutations, a conundrum is termed “Ohno’s dilemma” [3,4]. Scientists have attempted to test Ohno’s hypothesis, including the direct experimental tests [5]. However, despite close to 100 years of research, it remains an area of active research with many unanswered questions remaining about the molecular mechanisms by which gene duplication provides the raw material for evolution [6].

The fate of duplicated genes can vary and include loss, subfunctionalization, and neofunctionalization. The most common fate of duplicated genes is loss [7]. In most cases, gene loss is predicted to occur due to functional redundancy arising from gene duplication [8]. An interesting example from *Arabidopsis thaliana* (Arabidopsis) demonstrates that without gene loss, Arabidopsis should contain ~10 times the number of genes that it contains due to genome duplication events that have occurred during the evolution of this species from the common ancestor of land plants [9]. Although extensive gene loss is the most prominent outcome following duplication, genetic redundancy is a common feature in the genomes of higher organisms [8]. Duplicated genes are retained through mechanisms that are not mutually exclusive, including subfunctionalization, neofunctionalization, gene balance, genetic drift, genetic redundancy, paralog interference, and retention by increased gene dosage. Several models, including subfunctionalization, explain that duplicates can be retained by selection acting on existing functions. Driven by genetic drift, subfunctionalization occurs when each daughter copy of the duplicated gene assumes part of the function of the parental gene [10]. All copies are retained as the pre-duplicated functions become portioned [11,12]. Models supporting gene retention following subfunctionalization include the division of gene expression [11,13,14] and the specialization of the proteins encoded by the genes [15]. Duplicates can also be retained when genes assume a novel function. In neofunctionalization, one gene retains the ancestral function, whereas the paralog assumes a novel function. Both genes are retained if the novel function is beneficial and leads to better fitness. Examples of neofunctionalization in humans include eosinophil-derived neurotoxin (EDN) eosinophil cationic protein (ECP) genes [16], and red- and green-sensitive opsin genes [17]. In plants, examples include the B-class MADS-box transcription factors involved in determining organ identity in floral structures [18].

Gene duplications are common in genome evolution [7] and comparative genomic analysis provides the most data on the evaluation of duplicate genes [19]. Several recent studies have examined gene duplication in plants, demonstrating different models of gene duplication [9,20]. This includes whole-genome duplication (WGD) and subgenomic duplication events [9]. Examples of subgenomic duplication events in plants include retroduplication, tandem duplication, segmental duplication, and duplication mediated by transposable elements [9]. These studies also show that plants have undergone more frequent gene duplications than other eukaryotes, including more recent WGD events [9]. Retention of polyploids also occurred more frequently in plants [9]. Polyploid plants have more vigor than diploid plants, including increases in biomass, photosynthesis, and disease resistance [21]. As a result of WGD and polyploidy, plants have higher genome diversity compared to other eukaryotes, including more significant variations in genome size ranging from ~63 Mb to 150 Gb in dicots [9]. This contrasts with the variation in genome size from ~1.6 Gb to 8 Gb in animals [9]. In plants, the best-studied model of the diploid plant Arabidopsis has undergone at least two WGD events [22], with ~65% of Arabidopsis genes estimated to be duplicated (16,574 duplicated genes/25,498 total genes) [23]. Recent studies have looked in detail at the duplicated genes in Arabidopsis, including *EXOV* [24], *AT-HOOK MOTIF CONTAINING NUCLEAR LOCALIZED 15 (AHL15)* [25], and coat protein complex II (COPII) [26].

Transcription factor families are an example of the different outcomes of gene duplication events between plants and other eukaryotes. About 45% of plant transcription factors belong to gene families common to eukaryotes; however, most of these transcription factor families have a higher expansion rate in plants than in animals [27]. Arabidopsis has several examples of transcription factor families with multiple paralogs compared to its animal and fungal counterparts [28]. This includes, but is not limited to, the transcription factor family nuclear factor Y (NF-Y). However, the basis for gene retention after gene copy number expansion in NF-Y is virtually unknown. In general, the expansion of transcription factor families in plants (paralogs) also remains elusive.

Here we summarize the biological function of Arabidopsis NF-Y and focus on recent literature demonstrating that NF-Y expansion can be used as a model to study the gene expansion and retention of plant transcription factor families and plants in general.

## 2. Nuclear Factor Y (NF-Y) Transcription Factors

**Figure 1:**
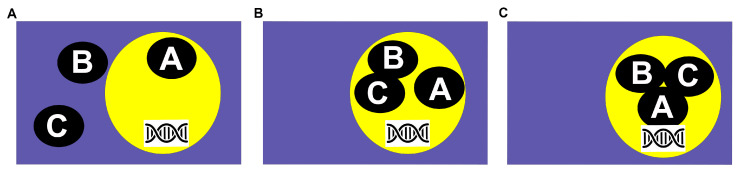
The NF-Y assembles in a stepwise manner. (**A**) NF-YA is localized to the nucleus (yellow circle), NF-YB moves freely between the nucleus and cytosol, and NF-YC is localized to the cytosol (blue rectangle). (**B**) To initiate gene transcription, the NF-YB/NF-YC dimerizes in the cytosol, moves to the nucleus, and binds the NF-YA. (**C**) Once assembled, the NF-Y trimer binds DNA to sequence-specific CCAAT elements.

NF-Y, also called the CCAAT-binding factor (CBF) or heme activator protein (HAP), is a transcription factor family found in all eukaryotes. NF-Y plays an essential role in eukaryotes as the loss of function of subunits is lethal to embryos [29]. The NF-Y transcription factor is a trimeric complex comprising three independent protein families: NF-YA, NF-YB, and NF-YC [30,31]. Plants have multiple independent NF-YA, NF-YB, and NF-YC subunits in contrast to other eukaryotes, which have one subunit of each NF-Y. Most information on NF-Y assembly and DNA binding comes from animal literature. NF-Y is highly conserved in eukaryotes [32], allowing plant biologists to extrapolate data from animal systems where NF-Y has been extensively studied. Studies in animal systems have shown that NF-YA is localized to the nucleus, NF-YB can move freely between the nucleus and cytosol, and NF-YC is localized to the cytosol [33,34]. NF-YB/NF-YC dimerizes in the cytosol, moves to the nucleus, and binds the NF-YA [35]. Once assembled, the NF-Y trimer binds DNA to sequence-specific *CCAAT* elements. NF-YA dictates sequence specificity, while the NF-YB/NF-YC dimer stabilizes the complex (Figure 1) [31,36]. Mutational analysis in yeast has identified amino acids that are essential for NF-YA to make physical contact with in the *CCAAT* boxes, and mutations of any single amino acid led to the loss of interaction [37]. *CCAAT* boxes are ubiquitous and present in as much as 30% of eukaryotic promoters [38]. Once bound, NF-Y can act as a positive or negative regulator of target genes.

## 3. The NF-Y Transcription Factors Have Expanded in Plants

Transcription factor family expansion is more common in plants compared to other eukaryotes [27]. An example is the *MYB* family of transcription factors. Arabidopsis has ~190 members; however, only a few are found in animals and yeast, precisely six in *Drosophila melanogaster* and ten in *Saccharomyces cerevisiae* [27]. Interestingly, NF-Y has undergone a significant expansion in plants. Animals, like humans, mice, and fruit flies, have one subunit each [37]. Fungi also only have one of each subunit; however, they contain a fourth transcription-activating subunit [39]. In contrast to animals and fungi, Arabidopsis has ten subunits of each (ten NF-YA, NF-YB, and NF-YC), totaling 30 subunits. Theoretically, the 30 subunits in Arabidopsis can produce 1000 unique DNA-binding NF-Y complexes, leading to enormous combinational complexity. Previously, in Arabidopsis, the number of NF-Y subunits was estimated to be ten NF-YA, 13 NF-YB, and 13 NF-YC subunits [40]. However, three members, each from NF-YB and NF-YC, were found to be homologs of *NC2* and *Dpb3/Dpb4*. NF-Y was reclassified to include ten NF-YA, 10 NF-YB, and ten NF-YC subunits [41], which remains the currently accepted classification.

The expansion seen in Arabidopsis also holds for other sequenced plant species. We have summarized the number of NF-Y subunits from plant species, including recently sequenced species, in Table 1. The table demonstrates that animals and fungi have one subunit of each NF-Y. In contrast, plants have multiple subunits. For example, the results show the number of NF-Ys to be 36 in *Brachypodium distachyon* [42], 37 in *Triticum aestivum* (wheat) [43], 33 in *Juglans regia* (walnut) [44], 41 in *Solanum tubersum* (potato) [45], 24 in *Prunus persica* (peach) [46], 34 in *Vitis vinifera* (grape) [47], 22 in *Citrus sinensis* (sweet orange) [48], 34 in *Orysa sativa* (rice) [49], 33 in *Sorghum bicolor* (Sorghum) [50], 19 in *Citrullus lanatus* (watermelon) [51], 52 in *Populus trichocarpa* (Populus) [52], 60 in *Medicago sativa* (alfalfa) [53,54], 27 in *Petunia hybrida* (Petunia) [55], 25 in *Ginkgo biloba* [56], 51 in *Saccharum* spp. (sugarcane) [57], 63 in *Nicotiana tabacum* (tobacco) [58], 27 in *Cucumis sativus* (cucumber) [59], and 108 in *Brassica napus* [60]. The data from Table 1 demonstrate the expansion of NF-Y in the plant lineage, which leads to the question: what are the biological functions of NF-Y and do they give indications of its expansion in plants?

## 4. Biological Functions of Plant NF-Y

The biological functions of NF-Y have been extensively studied in animals, partly due to their role in cancer development [61]. Significant advances have also been made in elucidating the biological functions of plant NF-Y in recent years. The best-studied biological function of NF-Y is its role as a positive regulator of photoperiod-dependent flowering. NF-Y interacts with the transcription factor, CONSTANTS (CO), to regulate the promoter of the gene *FLOWERING LOCUS T* (*FT*) [62,63,64,65,66,67,68,69]. Studies have also shown a mechanism for the epigenetic regulation of *FT* by an NF-Y/CURLY LEAF (CLF) protein complex [70]. NF-Y also regulates flowering via the *SUPPRESSOR OF OVEREXPRESSION OF CONSTANS 1 (SOC1)* promoter by binding together with Teosinte Branched 1/Cycloidea/Proliferating Cell Factor (TCP7) and REPRESSOR OF ga1-3 (RGA) [71]. NF-Y is also involved in embryogenesis [72,73,74,75,76,77], photomorphogenesis [78,79,80,81,82,83,84], seed dormancy and germination/abscisic acid (ABA) responses [73,85,86,87,88,89,90], drought tolerance [91,92,93,94], heat stress responses [90,95], freezing resistance [96], the salt stress response [89], unfolded protein response [97], gibberellic acid (GA) signaling [71,88,98], jasmonate signaling (JA) and disease resistance [99,100], brassinosteroid (BR) biosynthesis and signaling [101], root growth [102,103], and primary metabolism [104]. Table 2 summarizes our current understanding of the biological functions of Arabidopsis NF-Y and includes the Arabidopsis NF-Y subunits that play a role in biological functions, the promoters bound by the NF-Y during the process, and the protein–protein interaction partners. Supplementary Table S1 provides references for the data in Table 2. The findings demonstrate that NF-Y is involved in and coordinates several developmental processors. The common themes among its biological functions are flowering regulation, embryogenesis, stress response, and hormone signaling.

Although most research on the biological functions of the NF-Y has been performed on Arabidopsis, recent studies have shown that NF-Y plays a similar role in other plant species. Recent studies show that NF-Y plays a role in flowering time regulation [105,106,107,108,109,110], embryogenesis [111,112], seed dormancy and germination/abscisic acid (ABA) responses [113,114], drought tolerance [115,116,117,118,119,120,121,122], root growth [114], primary metabolism, and disease resistance [123,124] in many plant species, including economically important species. NF-Y also plays a role during nodulation in legumes [125]. Economically speaking, flowering time, nitrogen allocation, disease resistance, and stress adaptations, including drought stress adaptations and nodulation, are roles that have the potential for manipulation. For example, the overexpression of *NF-YA5* and *NF-YB2* has led to drought tolerance [91,92]. In the case of NF-YB2, the results were directly applicable to field-grown corn under drought conditions [92].

## 5. Regulation of Temporal and Spatial Gene Expression May Have Influenced the Retention of the Duplicated NF-Y

Controlled temporal and spatial gene expressions have been shown to influence the retention of duplicated genes through subfunctionalization [11,126]. The retention of multiple NF-Ys following gene duplications suggests this may be true for this transcription factor family. Extensive studies have been previously conducted on the expression pattern of NF-Y [40]. The promoter regions of each NF-Y were fused with a *GFP: GUS* reporter gene, and gene expression was studied in roots, rosettes, flowers, embryos, seed coats, and dark- vs. light-grown plants [40,85,87]. The expression of NF-Y genes varied in the different tissues studied, indicating that variations in gene expression may have contributed to the retention of duplicated genes.

Temporal and spatial expression patterns are closely related to biological functions. For example, expression patterns have been used to identify NF-Y subunits involved in a biological process. Gene expressions in ~10-day-old leaves can indicate a role in regulating CO-mediated photoperiod flowering time. Three NF-YC subunits (NF-YC3, NF-YC4, and NF-YC9) involved in regulating this process were identified using this approach, where each gene showed the expected temporal and spatial expression [127]. Interestingly, some NF-Y subunits with similar expression patterns show functional redundancy. *NF-YB2* and *NF-YB3* are expressed in ~10-day-old leaves and regulate photoperiod-dependent flowering [40]. Single mutations of these genes do not show a late flowering phenotype. However, the *nf-yb2 nf-yb3* double mutant shows the late flowering phenotype [68]. A similar phenomenon is seen with the three NF-YC subunits that regulate flowering. The *nf-yc3 nf-yc4 nf-yc9* triple mutant shows a late flowering phenotype not observed with the single mutants [127]. Although not the focus of this paper, analyzing spatial and temporal expressions is critical to explaining the retention of duplicated NF-Ys.

## 6. The Arabidopsis NF-Y Transcription Factors Can Be Classified into Ancestrally Related Subclasses

Phylogenetic analysis demonstrates that NF-Y can be divided into ancestrally related subclasses, as previously published [40]. Recently, the Arabidopsis NF-YB family was shown to be divided into four distinct ancestrally related subclasses based on their phylogenetic relationships [69]. Arabidopsis NF-YB proteins consistently showed clear paring to a recently diverged paralog. For example, NF-YB2/NF-YB3 and NF-YB6/NF-YB9 showed constant paring. Adding other monocots and dicot plant species to the phylogenetic analysis further supported the classes [69]. However, the placement of two members, NF-YB1 and NF-YB7, was less consistent and poorly supported, with bootstrap values below 70%. These two subunits were based on phylogenetic and functional relationships. In summary, the four clades of NF-YB included Class I: NF-YB1/NF-YB8/NF-YB10; Class II: NF-YB2/NF-YB3/NF-YB7; Class III: NF-YB4/NF-YB5, and Class IV: NF-YB6/NF-YB9.

**Figure 2:**
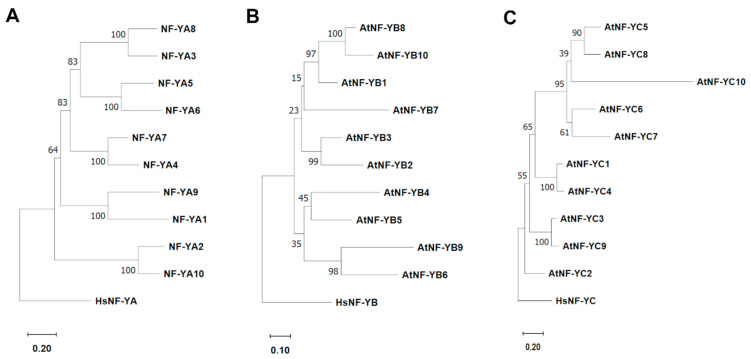
Based on phylogenetic relationships, Arabidopsis NF-Y proteins can be classified into ancestrally related subclasses. A neighbor-joining phylogenetic tree of Arabidopsis (**A**) NF-YA, (**B**) NF-YB, and (**C**) NF-YC, full-length proteins was constructed in MEGA11. Reliability values at each branch represent bootstrap samples with 2000 replicates. At, *Arabidopsis thaliana*; Hs, *Homo sapience*.

Here we analyzed the phylogenetic relationships of NF-YA, NF-YB, and NF-YC. The phylogenetic analysis was conducted using the full-length protein of each Arabidopsis NF-Y. The *Homo sapience* full-length proteins for the respective NF-Y subunit were used to root each tree. Phylogenetic relationships were examined using the neighbor-joining method with 2000 bootstrap replications in MEGA11 [128]. The TAIR accession number for each NF-Y member is provided in Supplementary Table S2. Our results show that all three subunits, NF-YA, NF-YB, and NF-YC, can be classified into ancestrally related subclasses (Figure 2). NF-YA had precise placement of each subunit into five classes, with NF-YA1/NF-YA9, NF-YA2/NF-YA10, NF-YA3/NF-YA8, NF-YA4/NF-YA7, and NF-YA5/NF-YA6 being placed in the same subclass. The bootstrap values for the placements were high, often 100%, giving high confidence in the placement of each member. As previously published, the placement of NF-YB members into subclasses was strongly supported in some cases and weakly supported in others [69]. Strong bootstrap values supported the placement of NF-YB2/NF-YB3, NF-YB6/NF-YB9, and NF-YB8/NF-YB10 into subclasses. NF-YB4/NF-YB5 were placed in one subclass, although the placement was poorly supported with low confidence. As previously published, the placement of NF-YB1 and NF-YB7 was least supported [69]. For the precise placement of NF-YC, high bootstrap scores were seen with NF-YC1/NF-YC4, NF-YC3/NF-YC9, and NF-YC5/NF-YC8. NF-YC6/NF-YC7 were also placed in a subclass with a 61% bootstrap score. Placement of NF-YC2 and NF-YC10 was least supported. Our data are consistent with previous publications [40]; the strongly supported placements for each NF-Y are consistent, whereas the weakly supported placements are placed differently based on the sequences, such as in full-length vs. conserved regions or the phylogenetic analysis methods utilized.

The placement of NF-Y members into ancestrally related subclasses allows us to study the similarities and differences between the members in these subclasses and examine whether these placements hold clues to gene duplication and retention. In some cases, the members of a class demonstrated apparent functional conservation. For example, NF-YB6 and NF-YB9 were consistently placed in the same class, and both members play an essential role in embryo development [75]. Other examples are NF-YB2/NF-YB3 and NF-YC3/NF-YC9, respectively, which play a role in regulating photoperiod-dependent flowering [69].

Next, we constructed multiple sequence alignments (MSAs) of the NF-Y proteins to study whether the protein structures could further explain duplication and retention.

## 7. The Arabidopsis NF-Y Proteins Share Conserved Core Domains Flanked by Non-Conserved N- and C-Termini

Multiple sequence alignments (MSAs) of NF-Y have been extensively studied previously [40,41]. Here, we constructed an MSA of each Arabidopsis NF-Y to analyze the full-length protein sequences using MUSCLE within Geneious [129]. Structurally, the MSA of all three NF-Ys has conserved regions flanked by non-conserved regions (Supplementary Figure S1). NF-YA has a single highly conserved core domain flanked by non-conserved N- and C-termini. The conserved core domain contains amino acids that are conserved between animals and plants. These include the conserved arginine and histidine residue in the 272–280 region and adjacent GxGGRF motif that makes physical contact with the CCAAT box [130]. The nuclear localization signal and amino acids that make physical contact with the NF-YB/NF-YC dimer are also highly conserved (Supplementary Figure S4). The flanking regions of NF-YA are less conserved; however, all Arabidopsis NF-YAs have a characteristic glutamine and serine/threonine-rich N-termini [131]. NF-YA proteins also share a region of apparent homology with a family of proteins called the COL (CO and CO-LIKE). The core domain of the NF-YA and the CCT domain (CO, CO-LIKE, TOC1) of the COL proteins share a region of high similarity [40]. COL proteins, including CO, physically interact with the NF-YB/NF-YC dimer, demonstrating a functional significance of the apparent homology [132,133].

MSAs of NF-YB and NF-YC subunits also reveal a highly conserved core domain flanked by non-conserved N- and C-termini (Supplementary Figures S2 and S3). The NF-YB and NF-YC core domains contain histone fold domains (HFDs) related to H2B and H2A histone proteins, respectively [130]. The amino acids in NF-YB and NF-YC involved in dimer formation, interaction with NF-YA, and physical contact with DNA are highly conserved (Supplementary Figure S4). Mutations made in these conserved amino acids in animals were shown to lead to loss of interactions [35,134]. Interestingly, mutations based on this literature in Arabidopsis subunits NF-YB2 and NF-YC9, also lost interaction with NF-YA2 [67,135].

High conservation across eukaryotes and loss of function in mutations suggests that the conserved core/HFD domain would be restricted in its ability to diverge following gene duplication. On the other hand, the less-conserved N- and C-termini provide residues that can change. Recent literature suggests this may be true; studies on the NF-YB suggest that conserved HFD may be under pressure for purifying selection, whereas the non-conserved N- and C-termini may be under diversifying selection [69].

## 8. The Study of Conserved and Non-Conserved Domains of Ancestrally Related Subclasses of NF-Y May Reveal Clues to Gene Duplication and Retention

Although gene duplication and retention have not been extensively studied in NF-Y, a recent publication sheds light on the topic [69]. The results of this study suggest that the N- and C-termini of NF-YB genes may be under pressure for diversifying selection and may provide clues to gene duplication and retention. Briefly, non-synonymous (Ka)/synonymous (Ks) substitution rates for NF-YB suggest that the conserved core HFD may be under pressure for purifying selections (negative), and the N- and C-termini may be under pressure for diversifying (positive) selection. Further, experimental analysis revealed that the N- and C-termini may have contributed to the retention of NF-YB members. The authors tested the ability of each of the ten Arabidopsis NF-YBs to substitute the function of NF-YB2/NF-YB3 during photoperiod-dependent flowering. Briefly, the experiments tested the ability of overexpression constructs for each *NF-YB* to rescue the late flowering phenotype of *nf-yb2 nf-yb3*. When only the conserved core HFM was cloned, most of the NF-YBs could rescue the phenotype, suggesting that the conserved core HFM alone is sufficient for photoperiod-dependent flowering. In contrast, the full-length proteins varied in their ability to rescue the phenotype, suggesting that the N- and C-termini may play role in the inability of some subunits to rescue the phenotype. Further studies were conducted by constructing a domain swap, where the three domains (HFM, N- and C-termini) of *NF-YB1* and *NF-YB2* were swapped. The results showed that the N- and C-termini may play a role in the differences in the flowering responses seen with the *NF-YB1* and *NF-YB2* full-length proteins to rescue. In summary, the data from the NF-YB suggest that the non-conserved N- and C-termini under pressure for diversifying selection may have allowed the duplicated genes to assume diverse biological functions, allowing their retention. The similarity of NF-YA and NF-YC, in terms of the ancestrally related subclasses and the protein’s primary structure, suggests that the observations made in NF-YB may hold for them.

## 9. Conclusions and Future Perspective

Here, we have summarized the current literature on NF-Y in plants. Several notable findings have been reported on these transcription factors in recent years, and we now have a broader understanding of their biological functions, summarized in Table 2. While advances have been made in understanding the NF-Y functions that have been known for several years, such as photoperiod flowering, embryogenesis, and stress responses, new biological roles have also emerged. The role of NF-Y in hormone signaling and responses is the most notable, including gibberellic acid, jasmonate, and brassinosteroid. Many recent publications have also focused on classifying and identifying NF-Y in non-model plant species, in which they play similar roles to the biological functions seen in Arabidopsis. Although many biological functions of the NF-Y have been identified in plants, the molecular mechanisms for these remain unknown and are an area for future research. The best-studied molecular mechanism, the role of NF-Y in photoperiod flowering, may provide a model for other biological processes. A clear understanding of molecular mechanisms could be particularly important for manipulating economically essential crops.

A recent publication provided significant insight into how the NF-Y transcription factor family expanded in plants. Although very little is currently known about the mechanisms of gene expansion and retention of NF-Y, or plant transcription factors in general, this paper’s findings suggest that the NF-Y can serve as a possible model for future studies. Although only the expansion of the NF-YB has been studied, we show here that the NF-YA and NF-YC have a similar pattern of expansion and can be used for potential future studies. In Figure 2, we show that all three subunits can be classified into ancestrally related subclasses. Further, all three subunits comprise the conserved core domains flanked by non-conserved N- and C-termini. The study of NF-YB demonstrates that the less conserved N- and C-termini may be under pressure for diversifying (positive) selection, while the conserved core HFD region may be under purifying selection (negative). Due to the similarity of NF-YA, and particularly the NF-YC protein, to NF-YB, similar results may be obtained with these two members and therefore warrant further research.

In summary, our understanding of plant NF-Y, including the biological roles, molecular mechanisms, and gene expansion, has improved over the past few years. While many questions remain, we expect our understanding of NF-Y to increase, especially considering its role in regulating agriculturally essential traits in economically important crops.

## Figures and Tables

**Table 1:** The NF-Y transcription factors have expanded in the plant lineage compared to other eukaryotes. The table summarizes the number of NF-Y subunits, NF-YA, NF-YB, and NF-YC, respectively, identified in different species of animals, fungi, and plants. Note that while animals and fungi have one subunit of each NF-Y, plants have multiple subunits.

Species	Number of NF-YAs	Number of NF-YBs	Number of NF-YCs
Animals			
*Homo sapiens* (human)	1	1	1
*Mus musculus* (mouse)	1	1	1
*Drosophila melanogaster* (fruit fly)	1	1	1
*Caenorhabditis elegans*	2	1	1
Fungi			
*Saccharomyces cerevisiae* (yeast)	1	1	1
*Candida albicans*	1	1	1
*Schizosaccharomyces pombe*	1	1	1
*Cryptococcus neoformans*	1	1	1
Plants			
*Arabidopsis thaliana* (Arabidopsis)	10	10	10
*Brachypodium distachyon*	7	17	12
*Triticum aestivum* (wheat)	10	11	14
*Juglans regia* (walnut)	17	9	7
*Solanum tubersum* (potato)	10	22	9
*Prunus persica* (peach)	6	12	6
*Vitis vinifera* (grape)	8	18	8
*Citrus sinensis* (sweet orange)	6	11	5
*Orysa sativa* (rice)	11	11	12
*Sorghum bicolor* (Sorghum)	8	11	14
*Citrullus lanatus* (watermelon)	7	8	4
*Medicago sativa* (alfalfa)	9	26	25
*Petunia hybrida* (petunia)	10	13	4
*Ginkgo biloba*	7	12	6
*Saccharum* spp. (sugarcane)	9	18	24
*Nicotiana tabacum* (tobacco)	17	30	16
*Cucumis sativus* (cucumber)	7	13	7
*Brassica napus*	38	46	24

**Table 2:** Summary of the biological functions of Arabidopsis NF-Y. The table summarizes the Arabidopsis NF-Y subunits published to play a role in each biological function, promoters bound by the NF-Y complex, and the known protein–protein interaction partners. The common themes among its biological functions are flowering regulation, embryogenesis, stress responses, and hormone signaling.

Biological Function	Arabidopsis NF-Y Subunit Published to Play a Role	Promoters Bound by the Arabidopsis NF-Y Complex	Protein–Protein Interaction Partners in Arabidopsis
Photoperiod-dependent flowering and floral transition	NF-YA1, and 2 NF-YB2, 3, 7 NF-YC1, 3, 4, and 9	*FLOWERING LOCUS T (FT)* *SUPPRESSOR OF OVEREXPRESSION OF CONSTANS 1 (SOC1)*	CONSTANTS (CO) Teosinte Branched 1/Cycloidea/Proliferating Cell Factor (TCP7) Curly Leaf (CLF)
Embryogenesis	NF-YA1, 3, 5, 6, 8, 9, and 10 NF-YB6 (LIL), and 9 (LEC1) NF-YC2	*CRUCIFERIN C* (*CRC*) Auxin/indole acetic acid repressor19 (*IAA19*) *Sucrose synthase 2* (*SUS2*)	PHYTOCHROME-INTERACTING FACTOR4 (PIF4) bZIP67
Photomorphogenesis/hypocotyl elongation	NF-YA2, and 5 NF-YB6, and 9 NF-YC1, 3, 4, 7 and 9	Auxin/indole acetic acid repressor (*IAA6*), and *IAA19* *PHYTOCHROME-INTERACTING FACTOR 3-LIKE 1 (PIL1)* *AT5G02580* *CYCLING DOF FACTOR 5 (CDF5)* *Light-harvesting chlorophyll a/b binding proteins (Lhcb)*	Pirin 1(PRN1) PHYTOSCHOME INTERACTING FACTOR 4 (PIF4) Cryptochrome 2 (CRY2) HDA15 ACTIN-RELATED PROTEIN6 (ARP6) TIMING OF CAB EXPRESSION 1 (TOC1) PRR5
Seed dormancy and germination/abscisic acid (ABA) responses	NF-YA1–10 NF-YB2, 3, 6, and 9 NF-YC3, 4, and 9	*Abscisic Acid Insensitive 5 (ABI5)*	ABA RESPONSE ELEMENT BINDING PROTEINS/ABA BINDING FACTOR 1 (ABF1), 2, 3, and 4 RGA-Like 2 (RGL2)
Drought tolerance	NF-YA5 NF-YB1, 2, and 3 NF-YC3, 4, and 9		ABA RESPONSE ELEMENT BINDING PROTEINS/ABA BINDING FACTOR 3 (ABF3), and 4
Heat stress responses	NF-YA2 NF-YB3	*Heat Stress Transcription Factor A3 (HsfA3)*	DNA POLYMERASE II SUBUNIT B3-1 (DBP3-1)
Freezing resistance	NF-YC1	*Xyloglucan Endotransglucosylase/Hydrolase 21 (XTH21)*	
Salt stress response	NF-YA1	*Abscisic Acid Insensitive 3 (ABI3), and 5*	
Unfolded protein response (a stress response)	NF-YA4 NF-YB3 NF-YC2		bZIP28
Gibberellic acid (GA) signaling	NF-YA1, and 2 NF-YB2 NF-YC3, 4, and 9	*SUPPRESSOR OF OVEREXPRESSION OF CONSTANS 1 (SOC1)*	RGA-Like 2 (RGL2) REPRESSOR OF ga1-3 (RGA) GA-INSENSITIVE (GAI) BRAHMA (BRM)
Jasmonate signaling (JA) and disease resistance	NF-YA1 NF-YB2, and 3 NF-YC9		JASMONATE-ZIM DOMAIN 8 (JAZ8), 9, and 11
Brassinosteroid (BR) biosynthesis and signaling	NF-YC1, 3, 4, and 9	*BR6ox2*	BRASSINOSTEROID INSENSITIVE2 (BIN2)
Root growth	NF-YA2, and 10 NF-YB2		
Primary metabolism	NF-YC4		Qua-Quine Starch (QQS)

## Supplementary Materials

The following supporting information can be downloaded at: https://www.mdpi.com/article/10.3390/ijms26010038/s1.
