# Supplementary material for: Plant Nuclear Factor Y (NF-Y) Transcription Factors: Evolving Insights into Biological Functions and Gene Expansion
Source: Int J Mol Sci. 2024 Dec 24;26(1):38. doi: 10.3390/ijms26010038 (PMC11719662; doi:10.3390/ijms26010038)
Supplement: Supplementary file 1 [file ijms-26-00038-s001.zip › Supplementary Table S1.pdf]

Supplementary Table S1: Summary of the biological functions of Arabidopsis NF-Y with the list of published references for each function. The table summarizes the Arabidopsis NF-Y subunits published to play a role in each biological function, promoters bound by the NF-Y complex, the known protein-protein interaction patterns, and references.

**Biological function**	**Arabidopsis NF-Y subunit published to play a role**	**Promoters bound by the Arabidopsis NF-Y complex**	**Protein-protein interaction partners in Arabidopsis**	**Reference**
Photoperiod-dependent Flowering and floral transition	NF-YA1, and 2<br>NF-YB2, 3, 7<br>NF-YC1, 3, 4, and 9	*FLOWERING LOCUS T (FT)*<br>*SUPPRESSOR OF OVEREXPRESSION OF CONSTANS 1 (SOC1)*	CONSTANTS (CO)<br>Teosinte Branched 1/Cycloidea/Proliferating Cell Factor (TCP7)<br>Curly Leaf (CLF)	Kumimoto *et al.,* (2008)<br>Hou *et al.,* (2014)<br>Siriwardana *et al.,* (2016)<br>Liu et al.,<br>Shen *et al.,* (2020)<br>Li *et al.,* (2021)<br>Lv *et al.,* (2021)

Embryogenesis	NF-YA1, 3, 5, 6, 8, 9, and 10<br>NF-YB6 (LIL), and 9 (LEC1)<br>NF-YC2	*CRUCIFERIN C* (*CRC*)<br>Auxin/indole acetic acid repressor19 (*IAA19)*<br>*Sucrose synthase 2* (*SUS2*)	PHYTOCHROME-INTERACTING FACTOR4 (PIF4)<br>bZIP67	West *et al.,* (1994)<br>Kwong *et al.,* (2003)<br>Yamamoto *et al.,* (2009)<br>Fornari *et al.,* (2013)<br>Mu *et al.,* (2013)
Photomorphogenesis/hypocotyl elongation	NF-YA2, and 5<br>NF-YB6, and 9<br>NF-YC1, 3, 4, 7 and 9	Auxin/indole acetic acid repressor (*IAA6), and IAA19*<br>*PHYTOCHROME-INTERACTING FACTOR 3-LIKE 1 (PIL1)*<br>*AT5G02580*<br>*CYCLING DOF FACTOR 5 (CDF5)*<br>*Light-harvesting chlorophyll a/b binding proteins (Lhcb)*	Pirin 1(PRN1)<br>PHYTOSCHOME INTERACTING FACTOR 4 (PIF4)<br>Cryptochrome 2 (CRY2)<br>HDA15<br>ACTIN-RELATED PROTEIN6 (ARP6)	Warpeha *et al.,* (2007)<br>Huang *et al.,* (2015)<br>Tang *et al.,* (2017)<br>Zhang *et al.,* (2021)<br>Yan *et al.,* (2021)

			TIMING OF CAB EXPRESSION 1 (TOC1)<br>PRR5	Wang *et al.,* (2023)
Seed dormancy and germination/Abscisic acid (ABA) responses	NF-YA1 - 10<br>NF-YB2, 3, 6, and 9<br>NF-YC3, 4, and 9	*Abscisic Acid Insensitive 5 (ABI5)*	ABA RESPONSE ELEMENT BINDING PROTEINS/ABA BINDING FACTOR 1 (ABF1), 2, 3, and 4<br>RGA-Like 2 (RGL2)	West *et al.,* (1994)<br>Nelson *et al.,* (2007)<br>Li *et al.,* (2008)<br>Leyva-González *et al.,* (2012)<br>Li *et al.,* (2013)<br>Liu *et al.,* (2016)<br>Sato *et al.,* (2019)
Drought tolerance	NF-YA5<br>NF-YB1, 2, and 3<br>NF-YC3, 4, and 9		ABA RESPONSE ELEMENT BINDING PROTEINS/ABA BINDING FACTOR 3 (ABF3), and 4	Nelson *et al.,* (2007)<br>Li *et al.,* (2008)<br>Liu et al., (2010)<br>Shi et al., (2014)

				Hwang *et al.*, (2019)<br>Sato et al., (2019)
Heat stress responses	NF-YA2<br>NF-YB3	*Heat Stress Transcription Factor A3 (HsfA3)*	DNA POLYMERASE II SUBUNIT B3-1 (DBP3-1)	Nelson *et al.*, (2007)<br>Li *et al.*, (2008)<br>Leyva-González *et al.*, (2012)<br>Sato *et al.*, (2014)<br>Sato *et al.*, (2019)
Freezing resistance	NF-YC1	*Xyloglucan Endotransglucosylase/Hydrolase 21 (XTH21)*		Shi *et al.*, (2014)
Salt stress response	NF-YA1	*Abscisic Acid Insensitive 3 (ABI3), and 5*		Li *et al.*, (2013)
Unfolded protein response (a stress response)	NF-YA4<br>NF-YB3<br>NF-YC2		bZIP28	Liu *et al.*, (2010)

Gibberellic Acid (GA) signaling	NF-YA1, and 2<br>NF-YB2<br>NF-YC3, 4, and 9	*SUPPRESSOR OF OVEREXPRESSION OF CONSTANS 1 (SOC1)*	RGA-Like 2 (RGL2)<br>REPRESSOR OF ga1-3 (RGA)<br>GA-INSENSITIVE (GAI)<br>BRAHMA (BRM)	Hou *et al.,* (2014)<br>Liu *et al.,* (2016)<br>Zhang *et al.,* (2023)
Jasmonate signaling (JA) and disease resistance	NF-YA1<br>NF-YB2, and 3<br>NF-YC9		JASMONATE-ZIM DOMAIN 8 (JAZ8), 9, and 11	Lin *et al.,* (2024)<br>Li *et al.,* (2024)
Brassinosteroid (BR) biosynthesis and signaling	NF-YC1, 3, 4, and 9	*BR6ox2*	BRASSINOSTEROID INSENSITIVE2 (BIN2)	Zhang *et al.,* (2021)
Root Growth	NF-YA2, and 10<br>NF-YB2			Ballif *et al.,* (2011)<br>Sorin et al., (2014)
Primary metabolism	NF-YC4		Qua-Quine Starch (QQS)	Li *et al.,* (2015)
