# Supplementary material for: Plant Nuclear Factor Y (NF-Y) Transcription Factors: Evolving Insights into Biological Functions and Gene Expansion
Source: Int J Mol Sci. 2024 Dec 24;26(1):38. doi: 10.3390/ijms26010038 (PMC11719662; doi:10.3390/ijms26010038)
Supplement: Supplementary file 1 [file ijms-26-00038-s001.zip › Supplementary Table S2.pdf]

Supplementary Table S2. The TAIR accession number of each NF-Y member in Arabidopsis. The accession numbers were obtained from Arabidopsis.org.

**NF-YA Member**	**TAIR Accession Number**	**NF-YB Member**	**TAIR Accession Number**	**NF-YC Member**	**TAIR Accession Number**
NF-YA1	AT5G12840	NF-YB1	AT2G38880	NF-YC1	AT3G48590
NF-YA2	AT3G05690	NF-YB2	AT5G47640	NF-YC2	AT1G56170
NF-YA3	AT1G72830	NF-YB3	AT4G14540	NF-YC3	AT1G54830
NF-YA4	AT2G34720	NF-YB4	AT1G09030	NF-YC4	AT5G63470
NF-YA5	AT1G54160	NF-YB5	AT2G47810	NF-YC5	AT5G50490
NF-YA6	AT3G14020	NF-YB6	AT5G47670	NF-YC6	AT5G50480
NF-YA7	AT1G30500	NF-YB7	AT2G13570	NF-YC7	AT5G50470
NF-YA8	AT1G17590	NF-YB8	AT2G37060	NF-YC8	AT5G27910
NF-YA9	AT3G20910	NF-YB9	AT1G21970	NF-YC9	AT1G08970
NF-YA10	AT5G06510	NF-YB10	AT3G53340	NF-YC10	AT1G07980
