# Supplementary figures and images for: Plant Nuclear Factor Y (NF-Y) Transcription Factors: Evolving Insights into Biological Functions and Gene Expansion
Source: Int J Mol Sci. 2024 Dec 24;26(1):38. doi: 10.3390/ijms26010038 (PMC11719662; doi:10.3390/ijms26010038)

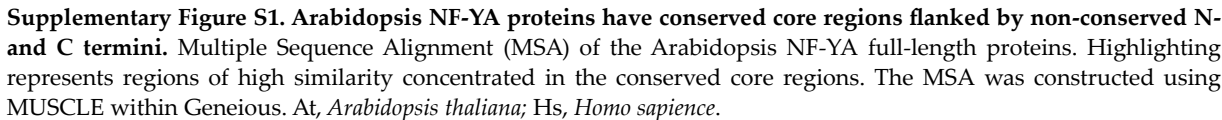

Supplement: Supplementary file 1 [file ijms-26-00038-s001.zip › Supplementary Figure S1.pdf]
