# Supplementary material for: Plant Nuclear Factor Y (NF-Y) Transcription Factors: Evolving Insights into Biological Functions and Gene Expansion
Source: Int J Mol Sci. 2024 Dec 24;26(1):38. doi: 10.3390/ijms26010038 (PMC11719662; doi:10.3390/ijms26010038)
Supplement: Supplementary file 1 [file Supplementary Figure S3.pdf › Supplementary Figure S3.pdf]

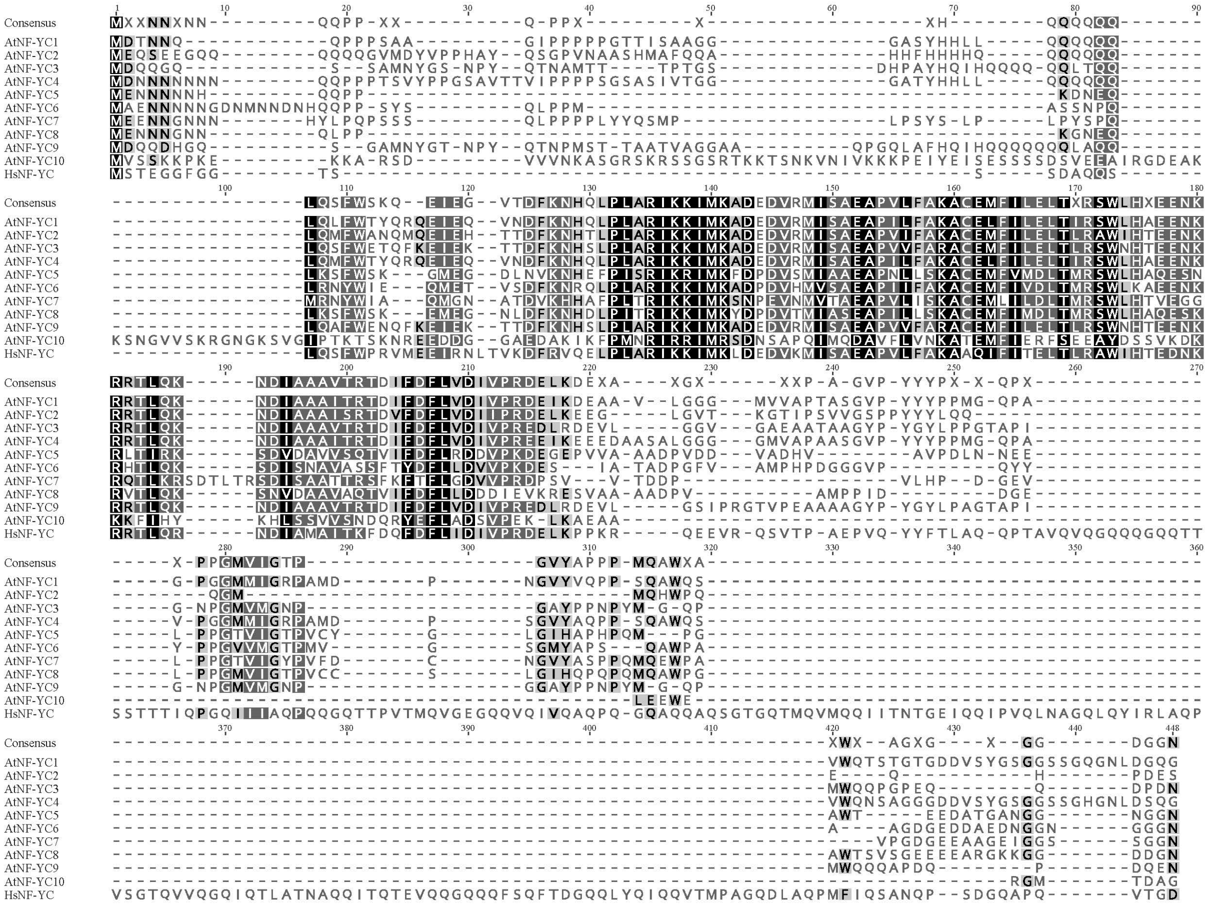

Supplementary Figure S3. Arabidopsis NF-YC proteins have conserved core regions flanked by non-conserved N- and C termini. Multiple Sequence Alignment (MSA) of the Arabidopsis NF-YC full-length proteins. Highlighting represents regions of high similarity concentrated in the conserved core regions. The MSA was constructed using MUSCLE within Geneious. At, *Arabidopsis thaliana;* Hs, *Homo sapience*.
