# Supplementary material for: Plant Nuclear Factor Y (NF-Y) Transcription Factors: Evolving Insights into Biological Functions and Gene Expansion
Source: Int J Mol Sci. 2024 Dec 24;26(1):38. doi: 10.3390/ijms26010038 (PMC11719662; doi:10.3390/ijms26010038)
Supplement: Supplementary file 1 [file Supplementary Figure S4.pdf › Supplementary Figure S4.pdf]

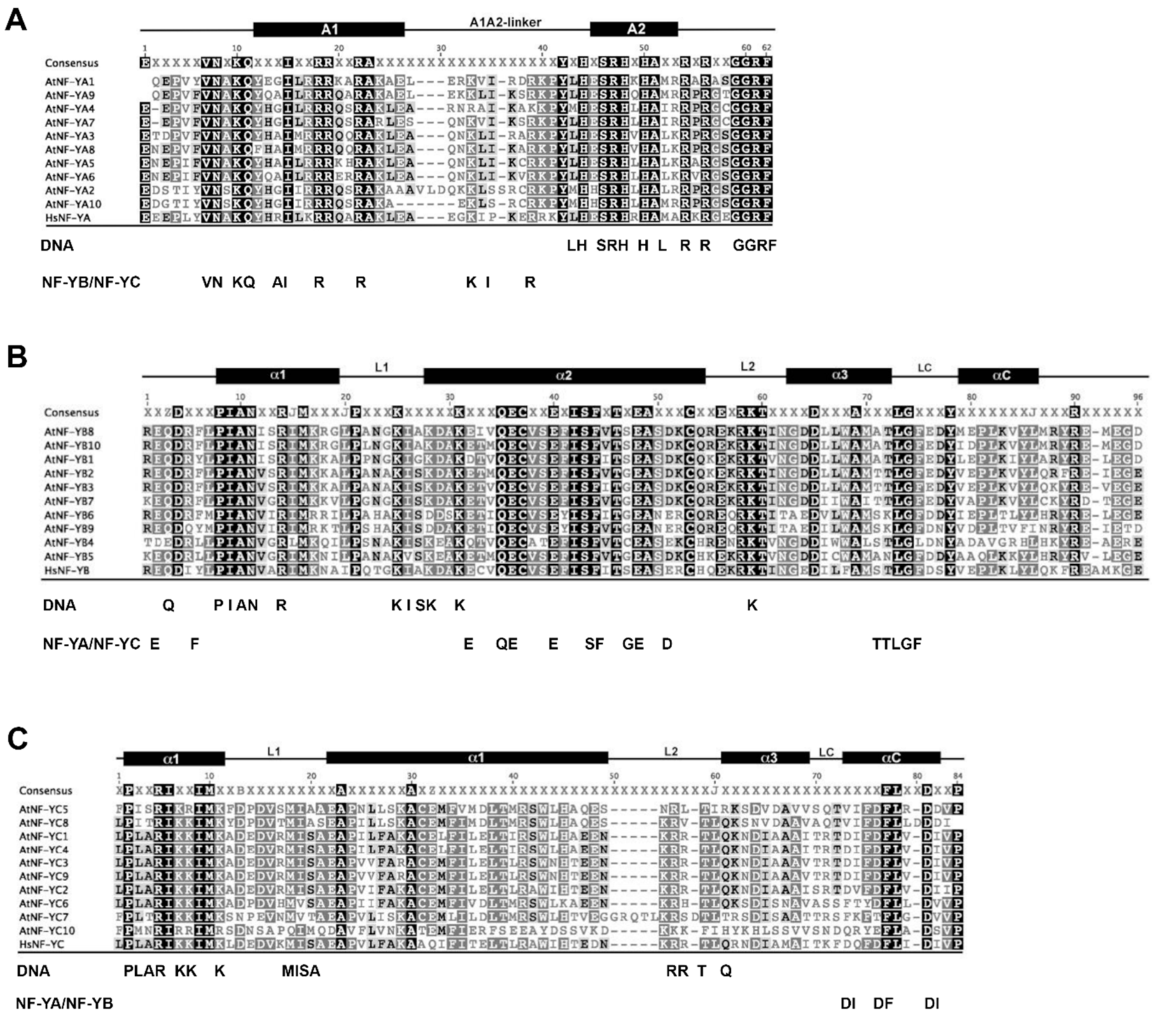

Supplementary Figure S4. Arabidopsis NF-Y proteins have conserved core regions required for interaction with DNA and NF-Y trimer formation. Multiple Sequence Alignment (MSA) of the core region of Arabidopsis (A) NF-YA, (B) NF-YB, and (C) NF-YC proteins. Highlighting represents regions of high similarity. Amino acids making physical contact with other NF-Y subunits and DNA are annotated, with reference to NF-YA3, NF-YB3, and NF-YC1[130]. The MSA was constructed using ClustalW within Geneious. At, *Arabidopsis thaliana;* Hs, *Homo sapience*.
